# Reducing Violence in Riyadh’s Emergency Departments: The Critical Role of Healthcare Providers

**DOI:** 10.3390/healthcare11060823

**Published:** 2023-03-10

**Authors:** Ahmed M. Al-Wathinani, Dennis G. Barten, Halah Al-Balawi, Sahar Al-Aqeel, Noora Al-Dulijan, Khulood Al-Ghamdi, Sarah Al-Shammari, Mohammad A. Alhallaf, Mohammad Aljuaid, Derrick Tin, Krzysztof Goniewicz

**Affiliations:** 1 Department of Emergency Medical Services, Prince Sultan bin Abdulaziz College for Emergency Medical Services, King Saud University, Riyadh 11451, Saudi Arabia; 2 Department of Emergency Medicine, VieCuri Medical Centre, 5912 BL Venlo, The Netherlands; 3 Department of Health Administration, College of Business Administration, King Saud University, Riyadh 11451, Saudi Arabia; 4 Faculty Disaster Medicine, Beth Israel Medical Center and Harvard Medical School, Boston, MA 02138, USA; 5 Department of Security Studies, Polish Air Force University, 08-521 Dęblin, Poland

**Keywords:** healthcare, violence, emergency department, aggressive behavior, violence prevention, healthcare providers

## Abstract

Emergency department staff are at high risk of experiencing violence and aggression from patients and visitors, which can have negative impacts on healthcare providers in the ED. The aim of this study was to explore the role of healthcare providers in addressing local violence in Riyadh EDs and investigate their preparedness for managing violent incidents. We used a descriptive, correlational design with survey methodology to collect data from a convenience sample of nurses, ED technicians, physicians, and advanced practice providers in Riyadh city’s EDs. To examine the associations, we used an analysis of variance (ANOVA) for unadjusted relationships and an analysis of covariance (ANCOVA) for adjusted associations. Measures included a demographic survey, and clinicians responded to an online survey. A total of 206 ED staff participated in the questionnaire, and 59% reported experiencing physical violence during an ED shift, with 61% of incidents being caused by relatives. Additionally, 32% of the participants witnessed workplace violence. Our findings revealed that male healthcare workers, physicians, and those working in the governmental sector were at the highest risk of experiencing violence. We also found a statistically significant association between the rate of patients seen in the ED and the frequency of assault (physical or verbal) in the ED. Our results suggest that the rate of workplace violence in Riyadh EDs is high, and more efforts are needed to protect the health and well-being of healthcare providers. Senior management should take a position against ED domestic violence and reinforce managerial and healthcare provider resources by adopting policies and procedures that protect healthcare workers’ safety. This study provides valuable insights into the nature and prevalence of violence in Riyadh EDs and highlights the critical role of healthcare providers in reducing violence in EDs.

## 1. Introduction

According to the International Labour Organization (ILO), workplace violence is defined as “any action, incident or behavior that withdraws from reasonable conduct in which a person is assaulted, threatened, harmed, injured in the course of, or as a direct consequence of, their work.” Emergency department staff are particularly vulnerable to workplace violence and violent and aggressive acts committed during violent incidents. Violent and aggressive acts committed by patients and visitors in emergency departments (EDs) remain a worldwide problem [1,2,3]. However, when initiating a career in healthcare, most healthcare providers do not anticipate that there may be concerns about their well-being every working day [4]. ED healthcare providers have reported experiencing a range of negative emotions, such as fear, confusion, anger, depression, guilt, embarrassment, helplessness, and disappointment due to violence [1,5]. The most frequently studied effects of such violence include a decline in job satisfaction and an elevated risk of burnout [6]. Moreover, instances of violent behavior encountered by ED staff are frequently not reported systematically. In qualitative research, employees have characterized workplace violence as a commonplace occurrence and an expected aspect of their job [7,8].

These risk factors for violent incidents in emergency departments include, for example, situations where patients or their family members may be in a highly emotional state, leading them to become aggressive or violent. Some patients may have mental health or substance abuse issues, which can increase their risk for violent behavior. Emergency department staff may be at increased risk for violence because they are often the first point of contact for patients in crisis. Some patients may feel frustrated or disrespected by the healthcare system, leading them to take out their anger on staff. Crowded and overworked emergency departments can also contribute to a high-stress environment, which can lead to incidents of violence [9,10,11,12].

Besides physical injuries, acts of violence (including verbal abuse) can result in severe negative effects on the mental health and well-being of healthcare workers, leading to a higher risk of burnout [3,13,14,15,16,17,18]. However, despite these risks, few violence prevention measures are currently in place, leaving employees feeling ill equipped to handle violent situations. To increase awareness of this issue, it is crucial to gain an understanding of the prevalence and impact of violence in emergency care.

Patient/visitor violence and aggression in the ED occur nearly every day with few evidence-based interventions that decrease the incidence. However, exposure to violence and aggression varies by position. Wong et al. observed that self-reported exposure to violent episodes was higher for technicians, nurses, and officers than for other healthcare professionals. Somani and colleagues [19] assessed the effectiveness of training in de-escalation and multicomponent interventions to decrease violence and hostility in the ED. There is a developing consensus that multicomponent interventions, including all stakeholders and the use of community advisory boards, are necessary to combat violence and aggression in the ED [9,19,20]

Patient and visitor violence and aggression are significant issues in healthcare settings that can threaten the safety and well-being of healthcare providers. There is a need for effective strategies to prevent and manage such incidents, which can help employees feel better prepared and more secure in their workplace. The aim of this study was to investigate the role of healthcare providers in addressing patient and visitor violence and aggression, as well as the attitude of their healthcare facilities toward these incidents. The novelty of our work lies in exploring these issues in the specific context of emergency departments in Riyadh, Saudi Arabia, where little research has been conducted on this topic. Our findings provide insights into the unique challenges faced by healthcare providers in this context and offer practical implications for improving the prevention and management of patient and visitor violence and aggression in emergency departments.

## 2. Materials and Methods

In this study, we utilized a descriptive, correlational design with a survey methodology to investigate the occurrence of patient/visitor violence and aggression against ED clinicians in Riyadh city’s EDs. Our sample consisted of nurses, ED technicians, physicians, and advanced practice providers who were conveniently recruited. To collect data, we used a demographic survey, and clinicians responded to an online survey. To evaluate the associations between different variables, we employed statistical analyses including analysis of variance (ANOVA) for unadjusted relationships and analysis of covariance (ANCOVA) for adjusted associations. We used correlation coefficients to measure the strength and direction of the relationship between variables. Specifically, we calculated Pearson’s correlation coefficient for continuous variables and Spearman’s rank correlation coefficient for ordinal variables. We also performed ANCOVA while controlling for potential confounding variables.

### 2.1. Sample and Procedures

To gather information about patient/visitor violence and aggression toward emergency department clinicians in Riyadh city, the present study utilized an online survey with a cross-sectional design. The survey was distributed to all ED staff, who received an informational flyer outlining the study’s purpose, procedures, participation conditions, and data management. Participants were encouraged to share the survey link with their emergency medicine colleagues using the snowball sampling technique. Follow-up reminders were sent to boost response rates. Data were collected from various EDs in Riyadh, Saudi Arabia, between September and November 2022. Study participants had to be at least 21 years old, work in EDs of hospitals or emergency services, and engage in direct interactions with patients and their families as part of their job. The sample included nurses, ED technicians, paramedics, physicians, and other healthcare providers.

### 2.2. Measures

In addition to collecting sociodemographic information, the online survey employed a comprehensive approach to capture data on various variables related to violent incidents. These included the frequency and nature of the incidents, the targets of aggression, and the measures taken to address it. The survey also assessed the stress and physical and psychological impact of such incidents, as well as the level of support from supervisors and colleagues. Additionally, the survey evaluated personal coping mechanisms, the ability to continue working, potential role changes, and mental health outcomes. Moreover, the survey explored the level of preparation provided by the workplace, and the availability of support after violent incidents, such as preventive measures, de-escalation training, reporting systems, and follow-up care. By covering these diverse measures, the survey aimed to provide a comprehensive evaluation of the experiences of emergency department clinicians who have encountered patient/visitor violence and aggression.

### 2.3. Statistical Tools

Statistical analyses were performed using SPSS v.28. The basic features of the data were described using frequency and percentage distributions. Spearman’s rank coefficient of correlation was used to assess the association between ordinal variables. Spearman’s rho is a nonparametric measure of rank correlation, denoted by the Greek letter ρ, which measures the strength and direction of the association between two ranked variables. This approach is appropriate for both continuous and discrete ordinal variables (Lehman, Ann; 2005). To examine the association between variables and sociodemographic factors, Pearson’s chi-squared test (χ^2^) was used. This statistical test is applied to sets of categorical data to test the independence of two variables, expressed in a contingency table. Independence means that knowing the value of the row variable does not change the probabilities of the column variable (and vice versa). Another way of looking at independence is to say that the row percentages (or column percentages) remain constant from row to row (or column to column). The strength of the correlation was evaluated using the following descriptors: very weak (0.0–0.19), weak (0.20–0.39), moderate (0.40–0.59), strong (0.60–0.79), and very strong (0.80–1.0).

### 2.4. Ethical Considerations

This study was approved by King Saud University Research Centre Institutional Review Board (Ref No.: KSU-HE-23-043), and informed consent to participate in this study was taken from each participant before answering the questionnaire.

## 3. Results

### 3.1. Descriptive Data

The sample size comprised 206 individuals, as 34 participants were excluded due to incomplete answers. In terms of demographic data, 53.9% of the survey responders worked for the governmental sector, 38.8% worked for the Ministry of Health (MOH), and 7.3% worked for the private sector. Physicians represented 47.6% of the sample, 27.7% were nurses, 14.1% were paramedics, and 10.7% were categorized as other healthcare providers. In terms of gender, 56.8% were male and 43.2% were female. Regarding experience, 38.8% had 1–5 years of experience, 26.2% had 6–10 years of experience, and 35% had more than 10 years of experience working in emergency departments (Table 1).

### 3.2. Local Violence in the Emergency Department

The participants responded to several questions about local violence in the ED; the highest percentage (27.2%) of the total sample examined 20–50 patients during their ED shifts. Our results showed that 58.7% of the participants had been physically assaulted during their work in the ED, 31.6% had witnessed another assault, and 9.7% had not witnessed or experienced assault. Of the total sample, 48.1% of the participants were physically or verbally assaulted 2–5 times during their work in the ED. The assault was perpetrated by patients’ family members or friends in 61.3% of the cases. The hospital administration responded to assaults in 35.5% of the cases, and 72.8% of the hospitals had clear policies and regulations to deal with assaults. Finally, assaults affected 41.7% of the total sample (physically or emotionally), and 84.5% indicated that violence in the ED could affect patient care (Table 2).

### 3.3. Frequency of Assault in Relation to Patient Exposure

The results of Spearman rho revealed the association between the rate of seeing patients during the emergency shift and the frequency of assault (physical or verbal) in the ED. The results indicated that there was a statistically significant association between the rate of patients seen during the emergency shift and the frequency of assault (physical or verbal) in the ED (r = 0.238, *p* < 0.01). This correlation coefficient indicated a positive association between the two variables, with higher patient volumes associated with a greater frequency of assault. While an r-value of 0.238 may not be considered a strong association in all contexts, it was considered statistically significant at the *p* < 0.01 level, indicating a meaningful relationship between these variables.

### 3.4. Being Assaulted in Relation to Demographic Variables

**Figure 1:**
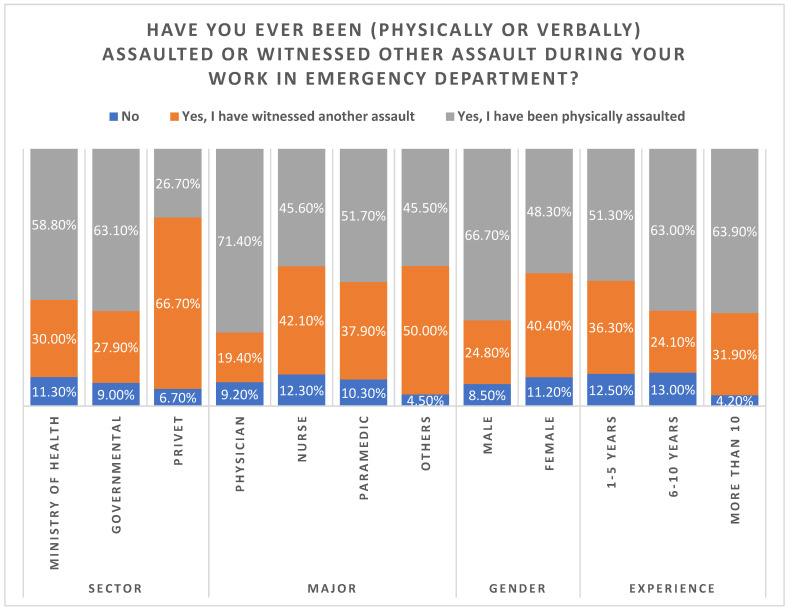
The rates of being assaulted or having witnessed other assaults during work in ED.

The test results of chi-square for an association between demographic variables and being assaulted (physically or verbally) or witnessing any assault while working in the ED indicated that there was a statistically significant association according to the sector, profession, and gender (*p* < 0.05). The governmental sector had a higher percentage of assaults during work in the ED (63.1%). Compared with other healthcare providers, physicians had a higher percentage of assault during their work in the ED (71.4%). Additionally, 66.7% of the male responders indicated that they had been physically assaulted, versus 48.3% of female responders. There was no statistically significant association between being assaulted (physically or verbally) or witnessing an assault while working in the ED and years of experience (*p* > 0.05) (Table 2, Figure 1).

### 3.5. Number of Assaults in Relation to Demographic Variables

**Figure 2:**
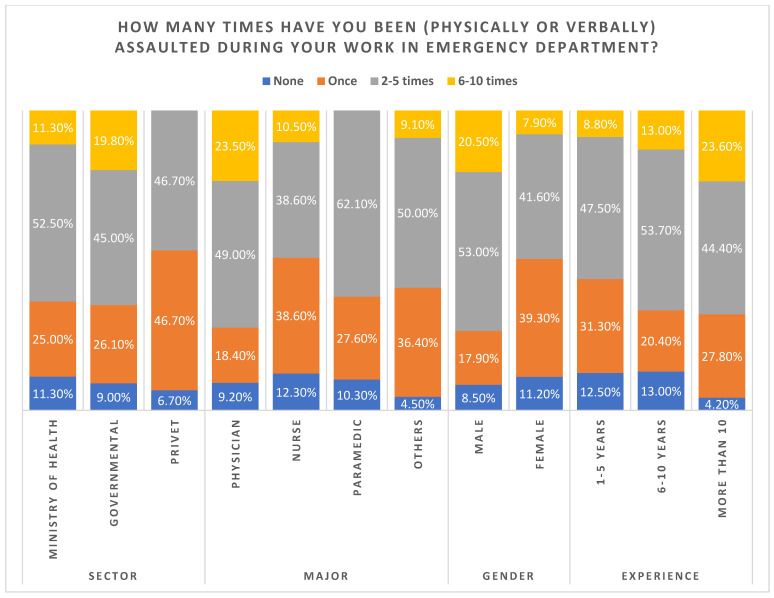
Number of times being assaulted in the emergency department.

Table 3 shows the test results of chi-square tests, which showed a statistically significant association between profession and gender (*p* < 0.05). Physicians reported a higher percentage of assaults (6 to 10 times during their work in the ED (23.5%)). In terms of gender, 20.5% of male healthcare workers were more likely to have experienced physical assault than their female counterparts (6 to 10 times for males versus 7.9% for females). Contrastingly, there was no statistically significant association between the number of assaults in the emergency room and sector or experience (*p* > 0.05) (Table 3, Figure 2).

## 4. Discussion

Numerous studies have thoroughly documented that healthcare providers are frequently exposed to physical and verbal abuse by patients and their relatives [3,10,11,12]. High prevalence rates of verbal and physical abuse were also observed in this survey among ED healthcare workers (90.3%).

This study also found a high prevalence of male participants who had been assaulted in the emergency department (53%), and most of them had 1–5 years of working experience in the ED (39%). In this study, there was a moderate association between being assaulted or witnessing an assault and being male, a physician, and working in the governmental sector. Nevertheless, according to different meta-analyses, gender, professional status, and closer interaction with patients and visitors may all contribute to female nurses encountering more physical abuse [3,14]. In a recent local study that examined the prevalence of healthcare workers’ exposure to violence in the Eastern Province of Saudi Arabia, it was found that health practitioners (46.9%) working in primary care centers were commonly exposed to different forms of abuse, including physical abuse. The study concluded by emphasizing that there was relatively little awareness and education on how to manage and report violence in healthcare institutions, thus stressing the need to establish a national program to track and prevent workplace violence [21].

Only a few review papers particularly addressed the extent of physical violence committed by patients or visitors in EDs against healthcare staff. According to a meta-analysis, 19.3% of medical professionals globally reported experiencing workplace violence committed by patients or visitors [3]. In our study, more than half of the healthcare workers had been assaulted either verbally or physically, 32% had witnessed someone else being assaulted, while 10% did not experience any domestic violence in the ED. In a cross-sectional multi-institutional study, it was found that emergency nurses (87.4%) were most frequently exposed to violence and (62%) of the violent encounters were perpetrated by the relatives of the patients [19]. Additionally, and as noted in two Saudi-based studies, one in Riyadh and one in Abha, some of the risk factors of violence in the Saudi healthcare setting included overcrowding, long wait times, culture and personality issues, understaffing, and most importantly, the lack of an encouraging environment for healthcare workers to submit official violence reports [22,23].

Several researchers have investigated the extent to which the experience of violent events at work may increase the chances of burnout, including fear, anger, and depression [1,6,20,24,25,26,27,28,29,30,31,32] A recent study found that non-physical violence, mainly verbal aggression, was associated with emotional exhaustion, cynicism, and reduced professional efficacy [15]. Likewise, the participants in our study agreed that the assault had an emotional or physical impact on them, and it also impacted patients’ treatment and care. On the other hand, hospital administrations responded to assaults for 36% of the total sample. Regarding hospital policies and regulations that deal with workplace violence, 73% had clear instructions and policies implemented in their healthcare system, which had a strong positive correlation (*p* = 0.01). However, only 39% of senior employees who responded to the survey indicated that the top management level had clearly taken a stand against violence.

The significant role of healthcare professionals and hospital policies regarding violent situations was one of the study’s important considerations. This study showed that in the local environment, male doctors and both sexes in other medical professions working in the ED suffered from an increased risk of verbal and physical abuse. Such situations may escalate and potentially cause harm. Thus, lower-level management employees may find it challenging to implement preventive measures or foster an open culture of conversation if the problem does not seem to be a priority for higher-level management. This deserves attention because preventing violence is a crucial management activity that greatly aids in establishing a secure work environment. This can be achieved by fostering an encouraging environment to report abuse incidents, conducting frequent risk assessments, and implementing preventative training and awareness programs.

According to a study conducted by Chen et al. in 2015, on workplace violence in Chinese hospitals, the prevalence of verbal and physical abuse was higher among female healthcare professionals [33]. Our study, on the other hand, found that male healthcare professionals were at a higher risk of experiencing verbal and physical abuse in the ED. This difference in results could be attributed to the cultural and contextual variations between China and Saudi Arabia.

In interpreting our results, it is evident that healthcare professionals and hospital policies play a significant role in managing and preventing violent situations. In Riyadh EDs, healthcare professionals, especially male doctors and both sexes in other medical professions, are at a heightened risk of experiencing verbal and physical abuse. Such incidents can quickly escalate and potentially cause harm, thus underscoring the need for preventive measures. It is important to note that lower-level managers may find it challenging to implement preventive measures or foster an open culture of conversation if preventing violence is not a priority for higher management levels. Therefore, it is crucial to establish a secure work environment by creating a culture of reporting abuse incidents, conducting frequent risk assessments, and implementing preventive training and awareness programs [34,35]. This approach could potentially reduce the prevalence of workplace violence in Riyadh EDs and create a safer work environment for healthcare professionals.

On a behavioral level, this entails providing behavioral training for staff members and managers that includes strategies and procedures, such as de-escalation instructions and self-defense methods. These can assist healthcare providers to improve their capability to handle dangerous or vital circumstances safely and competently [16,36,37,38]. Our hospital has taken steps to implement such training programs and has seen positive results in reducing the incidence of workplace violence. Additionally, it is worth mentioning that the Kingdom of Saudi Arabia, represented by the Ministry of Health’s Legal Affairs, has indicated that it will spare no effort to protect the country’s healthcare workers against abuse and will take the necessary legal measures to secure their rights. In fact, it is underlined in the Saudi Judicial System that “the right of all abused staff will be protected, indicating that verbal and physical abuse against health practitioners is a crime punished by law, with imprisonment up to 10 years and a fine up to one million riyals” [39]. Our hospital has also taken steps to work with local authorities to ensure that any incident of workplace violence is reported and investigated promptly and that perpetrators are held accountable for their actions. By implementing these solutions, our hospital has been successful in reducing the incidence of workplace violence in the emergency department. We believe that other hospitals facing similar challenges can benefit from our experiences and implement similar strategies to protect their healthcare workers and create a safer workplace for all.

## 5. Limitations

It is necessary to explain a few of our study’s limitations. Our research used an anonymous online survey. No causal connections could be found because of the cross-sectional design. The respondents were recruited for the survey using a link that was provided by Google Forms. As a result, response rates could not be determined. A selection bias may have resulted from this limitation. However, this made it possible to conduct the survey all around Riyadh city. Lastly, the survey of violent episodes in the ED in our cross-sectional study was recollected over a period of 3 months, which is quite short and can produce better results if it is extended for longer periods.

## 6. Conclusions

The rate of workplace violence in Riyadh EDs is high, and male healthcare workers, physicians, and those working in the government sector were at the highest risk of violence. Furthermore, there was an association with the rate of patients seen in the ED. We conclude that managing workplace violence is a difficult problem in the healthcare system and that management staff plays a crucial role to prevent, de-escalate, and deal with violent incidents. Future studies should assess how senior management should take a position against ED violence and reinforce the resources for managerial personnel and healthcare providers by the adoption of policies, procedures, and preventative training programs that protect healthcare workers’ health and well-being.

## Figures and Tables

**Table 1:** Demographic data of the sample study.

Demographic	Groups	N	%
Sector	Ministry of Health	80	38.8
	Governmental	111	53.9
	Private	15	7.3
	Total	206	100
Occupation	Physician	98	47.6
	Nurse	57	27.7
	Paramedic	29	14.1
	Others	22	10.7
	Total	206	100
Gender	Male	117	56.8
	Female	89	43.2
	Total	206	100
Years of experience in ED	1–5 years	80	38.8
	6–10 years	54	26.2
	More than 10	72	35.0
	Total	206	100

**Table 2:** Local violence in emergency department (n = 206).

Questions	Answers	N	%
What is the rate of patient seen by you during ED shift?	5–10	32	15.5
	10–20	38	18.4
	20–50	56	27.2
	50–100	47	22.8
	More than 100	33	16.0
Have you ever been (physically or verbally) assaulted or witnessed other assault during your work in emergency department?	No	20	9.7
	Yes, I have witnessed another assault	65	31.6
	Yes, I have been physically assaulted	121	58.7
How many times have you been (physically or verbally) assaulted during your work in emergency department? (n = 186)	None	20	9.7
	Once	56	27.2
	2–5 times	99	48.1
	6–10 times	31	15.0
Who committed the assault? “Please answer the most recent incident” (n = 186)	Patient	46	24.7
	Patient family member or friend	114	61.3
	Another visitor	4	2.2
	Colleague	4	2.2
	Unknown/other	18	9.7
Did your hospital administration respond to the assault?	Do not know	57	30.6
	No	63	33.9
	Yes	66	35.5
Does your hospital have a clear policy and regulation to deal with assault?	No	56	27.2
	Yes	150	72.8
Did the assault affect you (physically or emotionally)?	No	42	20.4
	Maybe	78	37.9
	Yes	86	41.7
In your opinion, does the violence in emergency department have an effect on patient’s care?	No	11	5.3
	Maybe	21	10.2
	Yes	174	84.5

**Table 3:** Association between demographic variables and being assaulted (physically or verbally) or witnessing any assault while working in the emergency department?

		Have You Ever Been (Physically or Verbally) Assaulted or Witnessed Other Assault during Your Work in Emergency Department?			Pearson Chi-Square	*p*-Value
		No	Yes, I Have Witnessed Another Assault	Yes, I Have Been Physically Assaulted		
In which health sector you are currently working?	Ministry of Health	11.3%	30.0%	58.8%	9.760	0.045
	Governmental	9.0%	27.9%	63.1%		
	Private	6.7%	66.7%	26.7%		
What is your profession?	Physician	9.2%	19.4%	71.4%	15.649	0.016
	Nurse	12.3%	42.1%	45.6%		
	Paramedic	10.3%	37.9%	51.7%		
	Others	4.5%	50.0%	45.5%		
What is your gender?	Male	8.5%	24.8%	66.7%	7.205	0.027
	Female	11.2%	40.4%	48.3%		
How many years of experience do you have in emergency department?	1–5 years	12.5%	36.3%	51.3%	6.282	0.179
	6–10 years	13.0%	24.1%	63.0%		
	More than 10	4.2%	31.9%	63.9%		

## Data Availability

The datasets used and/or analyzed during the current study are available from the corresponding author upon reasonable request.
